# Supplementary material for: The Spaces of Peer-Led Support Groups for Suicide Bereaved in Denmark and the Republic of Ireland: A Focus Group Study
Source: Int J Environ Res Public Health. 2022 Aug 11;19(16):9898. doi: 10.3390/ijerph19169898 (PMC9407788; doi:10.3390/ijerph19169898)
Supplement: Supplementary file 1 [file ijerph-19-09898-s001.zip › ijerph-1848168-supplementary.pdf]

Interview schedule: Focus Groups on peer-led Support Groups/activities for Bereaved by Suicide

Version 9.11.2020

Aim of the Focus Groups

We wish to establish focus groups where the participants share experiences with participating in support groups run by someone who has also lost a loved one to suicide (as opposed to groups run by professionals). We wish to gain insight into what helped and what did not and how or whether participation in the group affected the participants to establish a meaningful and satisfactory life, as defined by the individual participant, with the loss as a life condition.

Agenda for the Meeting

Duration: 150 minutes

Location: The online medium "Meetings" in Ireland, face to face meeting in Denmark

1. Introduction by moderator
2. Round of introduction
3. Dialogue in the group
4. Recapitulation
5. Closing

Ad 1) Introduction by Moderator (20 minutes incl. technical equipment/coffee etc.)

- Group framework: Confidentiality, respecting different opinions.
- To allow everyone a say, only one person should speak at a time.
- It can be a good idea to take notes if there is something you wish to remember afterwards.
- We are interested in your different experiences and perspectives in relation to participating in peer support groups/activities at FOSL/NABS, and how it has affected your everyday life.
- If you also have participated as moderator, organizer or similar, we would like to ask you to participate in this focus group as someone who has lost a loved one (and not as head of a group).
- There are no right or wrong answers/perspectives.
- We would like everyone to contribute to get as many different experiences, perspectives and nuances as possible on the individual subjects.
- The interview will be audio-recorded. Data will be treated confidentially and used for research purposes only.
- This is one of four focus groups. We subsequently intend to publish an article on the findings.
- All data will be anonymized; no one will be able to identify you or what you have talked about. But you may be able to recognize your words if used as a quotation in the article.
- We hope the knowledge generated here may show what is useful and what is not in peer support groups, and how it affects the participants' process of returning to/establishing a meaning and satisfactory everyday life with the loss is as a life condition.
- The dialogue/meeting will take no more than 150 minutes and will end at xx: xx. We will begin with short individual presentations followed by dialogue before closing the meeting.
- Does anyone have any questions?

Ad 2) Round of Introduction (30 minutes)

I would like to begin by asking each of you to introduce yourself with:

- First name
- Where are you from (region)?
- Age

- Who did you lose (son, daughter, spouse, brother, sister etc.)?
- Which groups/activities in FOSL/NABS did you participate in? For how long and how often?
- Please also describe any additional help you may have received from professionals, e.g. your GP or a psychologist

Ad 3) Group Dialogue (60 minutes)

Here are five questions that I would like you to discuss in relation to your individual experience with participating in the groups.

First, I would like to ask you to agree on the sequence of the questions.

I would also like to ask you to manage the time to allow time for all five questions:

1) How do you experience the activities in the association?
2) Which aspects of the form and content of the support groups/meetings do you consider important?
3) How is the social intercourse of the peer-led support groups different from the social intercourse of other situations in your everyday life?
4) In which way has participating in the various peer-led support groups/events affected your everyday life after the loss?
5) How do you experience that participating in the peer-led support groups/activities has helped your overall grief process?

Moderator:

The moderator may ask, ’What do the rest of you think when you hear this?’ Or: ‘Has anyone had the same experience?’ This is to add nuances/perspectives.

The moderator may also ask the participants whether there are situations where this does not happen. The aim here is to prompt critical thought and to establish what is not useful. E.g.: ’You say that it is important to be given a chance to speak. Have you been in situations where this was not possible? What did it mean to you?’

Ad 4) Recapitulation (20 minutes)

We have come to the recapitulation. Before we close the meeting I would like to ask you if there is something we have not touched upon today that you consider important with regard to understanding which aspects of the association’s groups are useful and which are not, and how you think the groups affect the process of returning to/establishing a meaningful and satisfactory everyday life after the loss?

Ad 5) Close (20 minutes)

Thank you for sharing your experiences. I would like to ask you what it is been like to participate in this focus group.

- If you would like the contact person from NABS/FOSL to call you later, please let me know or send me a text or an email.
- If you think of something in the next couple of days that you would like to add, please send me an email.
- Appointment on follow-up telephone call (to ensure that participants are referred to support if needed and possibility to elaborate on the focus group)
